# Nurse leadership in promoting and supporting civility in health care settings: A scoping review

**DOI:** 10.1111/jonm.13883

**Published:** 2022-11-15

**Authors:** Marianne Ota, Louisa Lam, Julia Gilbert, Danny Hills

**Affiliations:** ^1^ Institute of Health and Wellbeing Federation University Australia Mt Helen Victoria Australia; ^2^ School of Nursing, Midwifery and Paramedicine (VIC) Australian Catholic University Fitzroy Victoria Australia; ^3^ School of Public Health and Preventive Medicine Monash University Clayton Victoria Australia; ^4^ Institute of Health and Wellbeing Federation University Australia Brisbane City Queensland Australia

**Keywords:** civility, health care, nurse leaders, nursing, scoping review, workplace incivility

## Abstract

**Aim:**

This scoping review aimed to identify the existing evidence on how nurse leaders promote and maintain civility amongst nurses in health care settings.

**Background:**

Research on managing workplace incivility in nursing, a prevalent and concerning issue worldwide, recommends nurse leaders to command cultural change through strong leadership and civility interventions. However, there is very little empirical evidence summarizing and analysing how nurse leaders pragmatically achieve civility, and combat workplace incivility, in the health care setting.

**Evaluation:**

A scoping review was undertaken using the electronic databases CINAHL, Emerald Insight, MEDLINE, PsychINFO, PubMed and Scopus. Google Scholar was used to search for grey literature.

**Key issues:**

The eight studies included in this review describe how nurse leaders promote and maintain civility under four key themes: (1) creating a shared vision, (2) educating self and others, (3) fostering accountability and (4) providing support.

**Conclusion:**

The review provides an overview of commonly used strategies and actions that pragmatically promote and maintain civility in the health care setting by nurse leaders, while also highlighting areas of future research needed to strengthen the evidence base.

**Implications for Nursing Management:**

It is important for nurse leaders to gain an understanding of evidence‐based practices when addressing workplace incivility in order to address this prevailing problem for the future and safety of nurses moving forward.

## 1 BACKGROUND

Workplace incivility is internationally recognized as a prevalent and pressing issue in nursing, with clinical and workforce consequences clearly articulated across the literature over the past 20 years (Andersson & Pearson, 1999; El Ghaziri et al., 2021; Porath & Pearson, 2013). Strong links have been found between workplace incivility and burnout, resulting in poorer mental health amongst nurses and higher turnover intentions that exacerbate the global nursing shortage (Mikaelian & Stanley, 2016). In addition, workplace incivility can compromise patient safety and care delivery, eroding the success of organizations (Alquwez, 2020). Although professional nursing codes of conduct outline zero tolerance for bullying and harassment worldwide (Canadian Nurses Association, 2017; International Council of Nurses, 2021; Nursing and Midwifery Board of Australia, 2018), nurses continue to report higher levels of workplace incivility and bullying on a weekly basis than other health care professionals (Westbrook et al., 2021). Given the added pressures of the COVID‐19 pandemic on health services and workforces, achieving civility solutions that ensure the health and well‐being of both patient and provider is more imperative than ever.

A lack of clarity surrounds the definition of workplace incivility due to the synonymous use in the literature of similar but distinctly different terms like horizontal violence, lateral violence, bullying and unprofessional behaviour (Patel & Chrisman, 2020). Andersson and Pearson (1999), however, provided a foundational and widely accepted definition, namely, that workplace incivility comprises rude and unprofessional behaviours of low‐intensity and ambiguous intent that violate the acceptable norms of respect within an organization. Gossiping, spreading rumours, eye‐rolling and smirking threaten our intrinsic sense of self‐worth, giving way to feelings of powerlessness, anger and humiliation (Clark, 2017). When left unaddressed, symptoms such as headache, interrupted sleep, intestinal issues, anxiety, depression and feelings of stress may arise, potentially developing into post‐traumatic stress disorder or even precipitating suicidal intent (McPherson & Buxton, 2019). Unlike workplace aggression and bullying that involve clearly intended acts to harm others (Hills, 2018), workplace incivility may occur unintentionally through thoughtlessness or ignorance (Abolfazl Vagharseyyedin, 2015). According to Namie (2003), a 10‐point continuum of organizational disruption places workplace incivility between a 1 and 3, whereas bullying covers mild to severe interreference with a score of 4 to 9. Regardless of seniority or position within an organization, workplace incivility can occur at any level of employment (Schilpzand et al., 2016). In the end, workplace incivility entails a lack of regard for the feelings of others (Andersson & Pearson, 1999), that not only affronts the dignity of that person (Clark, 2017) but also erodes professional standards of practice (Meier et al., 2021), and threatens the values upholding the integrity of organizations (Leiter, 2013).

Civility, on the other hand, is the act of respecting others, especially in situations where disagreement or disparity may arise (Clark et al., 2022). According to Clark et al. (2022), civility occurs by modelling kindness and empathy in an intentional manner that seeks to understand differences and upholds common ground across all social contexts (Clark et al., 2022).

Authentic, resonant, servant and transformational leadership styles have widely been proposed to enable change to promote civility (Mikaelian & Stanley, 2016). Authentic leaders carry a strong sense of self and actively practice core values (Giordano‐Mulligan & Eckardt, 2019), whereas resonant leaders focus on building trusting relationships to ensure positive outcomes for their followers (Squires et al., 2010). Placing faith in their followers' abilities is a defining trait of servant leaders (Neubert et al., 2021), who focus on serving others. Conversely, transformational leaders foster growth in followers by fostering interpersonal relationships (Kaiser, 2017).

It has been widely recommended that nurse leaders address workplace incivility by commanding cultural change within organizations (Clark, 2019; Kavakli & Yildirim, 2022), namely, through strong leadership (Bagnasco et al., 2018; Crawford et al., 2019) and the delivery of civility interventions (Armstrong, 2018; Kile et al., 2019). However, little is understood regarding the manner in which nurse leaders set the tone for the culture, expectations and acceptable behaviours within their workplace (Kaiser, 2017; Porath & Pearson, 2013) or the effectiveness of civility interventions (Scott & Hills, 2021). According to Clark et al. (2011), increased workloads, poor relationships, poor work conditions, role ambiguity and a lack of knowledge in managing conflict contribute to the ability of nurse leaders to foster civility in the practice setting. These recommendations are often broad and problematic as they fail to provide evidence relating to the specific and effective strategies and actions that nurse leaders use to pragmatically achieve civility in practice. Currently, there is very limited empirical evidence uncovering how nurse leaders promote and maintain civility, and combat workplace incivility, in health care settings. Hence, given this challenging environment, there is a need to identify empirically tested strategies and actions to practically enhance the experiences of nurse leaders to intentionally foster civility for all nurses in the practice setting.

## 2 REVIEW METHODS

### 2.1 Aim

The aim of this scoping review is to identify the current evidence on the actions and strategies nurse leaders employ to promote and maintain civility in practice. Nurse leaders in this study are defined as any nurse working within a leadership or management role requiring the leading of nurses in teams.

### 2.2 Design

A scoping review was undertaken due to the developing nature of this research area (Munn et al., 2018). A scoping review not only provides clarification for future areas of research but also maps the current evidence to form an overview of knowledge (Peters et al., 2020).

### 2.3 Search methods

The authors utilized the Joanna Briggs Institute *Methodology for JBI Scoping Reviews* as a guide to inform this review (Peters et al., 2020).

The research question developed to guide this review is as follows:

*What is the existing evidence on nurse leadership in promoting and supporting a culture of civility in health care settings?*

The review utilized six databases including CINAHL, Emerald Insight, MEDLINE, PsychINFO, PubMed and Scopus. Google Scholar was employed to identify any grey literature. A librarian from Federation University Australia's Institute of Health and Wellbeing was consulted regarding the search terms and search strategy. Medical Subject Heading (MeSH) terms were also employed to enhance the search. The search terms included civil N5 behavio#r, OR civility, OR incivil*, OR uncivil*, OR rude*, AND nurs*, AND leader*, OR leading, OR manag*, OR supervis*, OR executive*, OR administrat*, AND clinical, OR hospital*, OR staff, OR personnel, OR provider*, OR worker*, OR professional*, NOT academi* AND faculty. The terms ‘bully’ and ‘bullying’ were omitted from the search as this form of workplace mistreatment is related to more intense acts of repetitive and systematic disrespect when compared with workplace incivility (Abolfazl Vagharseyyedin, 2015), although workplace incivility can be a precursor to bullying (Andersson & Pearson, 1999). Search terms were modified to meet the requirements of each database to ensure an accurate result.

### 2.4 Study selection

**FIGURE 1:**
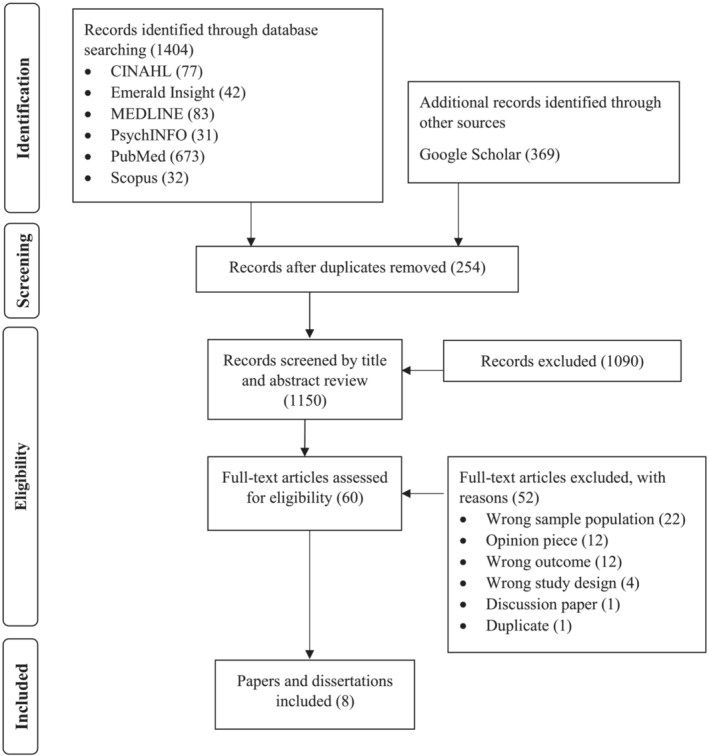
Search flow diagram

A total of 1404 abstracts were sourced across the databases. The review included primary qualitative and quantitative peer‐reviewed research reports, as well as grey literature. Review articles were excluded to avoid the repetition of knowledge. Reference lists were reviewed to identify additional reports. Abstracts were exported from the databases and Google Scholar into Endnote and subsequently uploaded to Covidence (Veritas Health Innovation, 2021) for appraisal by a team of three reviewers. Sources written in English and published between 2000 and 2021 were included in the search. Following the removal of 254 duplicates, titles and abstracts were then screened for relevance, resulting in 60 studies eligible for full‐text review. Both nurse leaders and nurses who were able to report on the strategies and actions employed by nurse leaders with regard to civility and workplace incivility, including staff nurses and new graduate nurses, were included. As civility is a broad term, studies were included that specifically reported on levels of experienced workplace incivility or civility. Studies exploring similar topics, such as workplace culture or respect, were excluded if there was no empirical evidence of the association between the practices of nurse leaders with workplace incivility or civility. Studies were also excluded if the research was conducted outside of a health care setting, such as an academic environment. After discarding 49 studies that did not qualify for inclusion, eight studies remained (Figure 1).

Studies were assessed for quality using the JBI Critical Appraisal Checklist for Analytical Cross‐Sectional Studies (Moola et al., 2017) and the JBI Critical Appraisal Checklist for Qualitative Research (Lockwood et al., 2020). In order to apply the checklists, the researchers determined acceptable scores as a team. Cross‐sectional studies were accepted with a score of 6 and above. The qualitative study was accepted with a score of 8 and above. Each researcher completed the checklists separately. No differences in scores were evident across the team.

### 2.5 Data extraction and charting

Data were extracted and organized into tables under relevant headings including authors, year of publication and study origin, study type, study design and aim, sample, sample size, leadership style, method, findings and limitations (Table 1). Each research study was carefully reviewed using the six‐step thematic approach described by Braun and Clarke (2021) to engage with the data and identify codes that were subsequently developed into key themes.

**TABLE 1:** Studies regarding how nurse leaders promote civility

Author(s), year and country	Type	Design and aim	Sample and speciality	Sample size	Leadership style	Direction of workplace incivility	Method	Findings	Limitations
Alkaabi and Wong (2019) Canada	Master of Science dissertation	Cross‐sectional To examine the relationship between new graduate nurses' experiences of manager incivility and their degree of trust in their managers.	New graduate nurses Medical‐surgical unit	1020	Authentic	From manager	Mailed survey consisting of the Authentic Leadership Questionnaire, Straightforward Incivility Scale and Trust in the Manager Scale. Descriptive statistics	Managers who practise authentic leadership are less likely to display workplace incivility, which hinders trust between the manager and new graduate nurses.	Self‐report surveys increase response bias. Data collection is limited due to being part of a larger project. Inferences of causality are limited due to covariance between variables and theory.
Clark et al. (2011) United States	Journal article	Qualitative To explore the perceptions of nurse leaders regarding civility in nursing education and practice, specifically in how to effectively address and foster civility amongst nurses.	Nurse leaders Speciality not reported	174	Not investigated	Unit‐level	Paper‐based survey consisting of 4 open‐ended questions distributed at a state‐wide nursing conference. Thematic analysis	Behaviours that promote civility: 1. Conduct meetings with nurses to develop a shared vision of a culture of civility. 2. Establish codes of conducts and policies with clear expectations of behaviours. 3. Deliver ongoing education related to conflict resolution, problem solving and respectful communication. 4. Deliver ongoing education related to practice preparedness. 5. Practise positive role‐modelling. 6. Focus on promoting a healthy practice environment. 7. Practice accountability for acceptable behaviours for both self and others. 8. Reinforce positive behaviours.	Small study conducted at a conference. Conceptual framework requires empirical testing.
Kaiser (2017) United States	Journal article	Cross‐sectional To examine the association between leadership style and nurse‐to‐nurse incivility, and to explore leader behaviours that impact staff relationships.	Staff nurses Speciality not reported	237	Authentic	From co‐worker	Online survey consisting of the Vannsimpco Leadership Survey and Nursing Incivility Scale. Parametric, correlation and regression analysis	Behaviours that promote civility: 1. Foster teamwork between staff and managers. 2. Be highly involved in day‐to‐day issues in the workplace. 3. Empower staff by asking for input and making decisions based on this feedback. 4. Show genuine interest in the development of staff and their abilities. 5. Have close relationships with staff. 6. Recognize and reward good work amongst staff. 7. Involve staff in the vision of the organization. 8. Enforce policies and procedures in daily workflow.	Constructs of leadership and workplace incivility are subjective. Survey not tested rigorously for psychometric properties.
Laschinger and Read (2016) Canada	Journal article	Cross‐sectional To examine how person–job fit and authentic leadership impact civility norms and how these norms affect incivility amongst co‐workers and resulting emotional exhaustion.	New graduate nurses Medical‐surgical unit	993	Authentic	From co‐worker	Mailed survey consisting of the Authentic Leadership Questionnaire, Areas of Worklife Scale, Civility Norms Questionnaire, Straightforward Workplace Incivility Scale, Co‐worker Incivility Subscale, Maslach Burnout Inventory and Emotional Exhaustion Subscale. Structural equation modelling	Authentic leaders develop civility norms for new graduate nurses by addressing the 6 areas of person–job fit. The 6 areas of worklife include workload, control, reward, community, fairness and values.	Includes only Canadian new graduate nurses. Limited causality due to cross‐sectional design.
Laschinger et al. (2014) Canada	Journal article	Cross‐sectional To examine a link between a positive leadership approach and workplace empowerment to workplace incivility, burnout and job satisfaction.	Staff nurses Medical‐surgical unit	1241	Resonant	From co‐worker	Mailed survey consisting of the Resonant Leadership Scale, Global Empowerment Scale, Workplace Incivility Scale, Maslach Burnout Inventory‐General Survey and Global Measure for Work Satisfaction. Structural equation modelling	Resonant leadership has a strong positive effect on workplace empowerment, which in turn has a significant negative effect on workplace incivility.	Limited causality due to cross‐sectional design. Common method variance bias may be present.
Lewis and Malecha (2011) United States	Journal article	Cross‐sectional To examine the impact of workplace incivility on staff nurses in terms of productivity and cost.	Staff nurses Operating room	2160	Not mentioned	From general environment, nurse supervisor and physician	Participants given option of completing mailed or online survey. Survey consisted of the Nursing Incivility Scale and Work Limitation Questionnaire. Content, construct and criterion validity	Nurses who perceive their managers' ability to effectively handle workplace incivility report lower levels of experienced workplace incivility.	Study conducted at one American organization.
Neubert et al. (2021) United States	Journal article	Cross‐sectional To investigate how nurse leaders prevent incivility by focusing on the needs of staff and encouraging a virtuous climate.	Staff nurses Speciality not reported	1485	Servant	Unit‐level	Online survey consisting of the Virtuous Behaviour Scale and Incivility Scale. Unit data were also used. Descriptive statistics and correlation	Servant leaders reduce workplace incivility by promoting virtuous practices and process that are accepted and copied by staff, which, in turn, promote a virtuous climate that signals the inappropriateness of workplace incivility.	Servant leadership may be influenced by a virtuous climate, not necessarily vice versa.
Smith et al. (2018) United States	Journal article	Cross‐sectional To determine whether workplace incivility between nurses is related to nurse work environment.	Staff nurses Critical care unit	233	Authentic	From co‐worker	Online survey consisting of the National Quality Forum‐endorsed Practice Environment Scale of the Nursing Work Index and Workplace Incivility Scale. Descriptive univariate analysis, correlational and linear regression	Staff nurses report less workplace incivility when there is a positive perception of the ability, leadership and support offered by the nurse manager.	Limited causality due to cross‐sectional design. Difficult to generalize findings as conducted at Magnet and Pathway to Excellence hospitals. Same‐source bias. Survey fatigue may have caused a low response rate.

## 3 RESULTS

### 3.1 Study characteristics

Of the eight studies included in the final review, three originated from Canada and five originated from the United States. No reports were included from the search of grey literature. With the exception of one qualitative study (Clark et al., 2011), the remainder of the included studies employed a cross‐sectional design. Sample populations constituted nurses from the same role, with one study including nurse leaders (Clark et al., 2011), two studies including new graduate nurses (Alkaabi & Wong, 2019; Laschinger & Read, 2016) and five studies including staff nurses (Kaiser, 2017; Laschinger et al., 2014; Lewis & Malecha, 2011; Neubert et al., 2021; Smith et al., 2018). Nurses reported working in medical‐surgical units for the majority of the reviewed studies, followed by the operating room (Lewis & Malecha, 2011) and critical care unit (Smith et al., 2018). However, three of the reviewed studies did not collect or report on nurses' areas of employment (Clark et al., 2011; Kaiser, 2017; Neubert et al., 2021). Online surveys were used in three studies (Kaiser, 2017; Neubert et al., 2021; Smith et al., 2018), and one study gave participants the option of completing the survey online or by mail (Lewis & Malecha, 2011). Mailed surveys questionnaires were distributed in three studies (Alkaabi & Wong, 2019; Laschinger et al., 2014; Lewis & Malecha, 2011), with one study distributing a paper questionnaire containing four open‐ended questions to nurses attending a conference (Clark et al., 2011).

Workplace incivility was most commonly measured using the Workplace Incivility Scale (Cortina et al., 2001). Both the Nursing Incivility Scale (Guidroz et al., 2010) and the Straightforward Incivility Scale (Leiter & Day, 2013) were used twice (Alkaabi & Wong, 2019; Kaiser, 2017; Laschinger & Read, 2016; Lewis & Malecha, 2011). Civility was measured in one study using the Civility Norms Questionnaire (Laschinger & Read, 2016; Walsh et al., 2012). Whereas the majority of participants from the included studies reported exclusively on workplace incivility experienced between co‐workers, two studies investigated unit‐level workplace incivility (Clark et al., 2011; Neubert et al., 2021), one included instigation from the manager (Alkaabi & Wong, 2019) and one included workplace incivility from the general environment, nurse supervisor and physician (Lewis & Malecha, 2011).

In terms of quality (Table 2), the majority of cross‐sectional studies were awarded six out of eight items. These studies represented the poorest quality overall as they failed to consistently identify confounding factors (Kaiser, 2017; Laschinger et al., 2014; Laschinger & Read, 2016; Lewis & Malecha, 2011; Smith et al., 2018). The qualitative study (Clark et al., 2011) was awarded 8 out of 10 items (Table 3), given that the influence of the researchers on the participants, and vice versa, was not identified, and there was no statement to locate the researchers culturally or theoretically.

**TABLE 2:** Quality assessment of cross‐sectional studies using the JBI Critical Appraisal Checklist for Analytical Cross‐Sectional Studies

Questions from checklist	Alkaabi and Wong (2019)	Kaiser (2017)	Laschinger and Read (2016)	Laschinger et al. (2014)	Lewis and Malecha (2011)	Neubert et al. (2021)	Smith et al. (2018)
1. Were the criteria for inclusion in the sample clearly defined? 2 Were the study subjects and the setting described in detail?	+	+	+	+	+	+	+
	+	+	+	+	+	+	+
3 Was the exposure measured in a valid and reliable way?	+	+	+	+	+	+	+
4 Were objective, standard criteria used for measurement of the condition?	+	+	+	+	+	+	+
5 Were confounding factors identified?	+	−	−	−	−	+	−
6 Were strategies to deal with confounding factors stated?	−	−	−	−	−	+	−
7 Were the outcomes measured in a valid and reliable way?	+	+	+	+	+	+	+
8 Was appropriate statistical analysis used?	+	+	+	+	+	+	+
Outcome	7/8	6/8	6/8	6/8	6/8	8/8	6/8

*Note*: The plus symbol ‘+’ indicates a ‘yes’ to the question. The minus symbol ‘−’ indicates a ‘no’ to the question.

**TABLE 3:** Quality assessment of a qualitative study using the JBI Critical Appraisal Checklist for Qualitative Research

Questions from checklist	Clark et al. (2011)
Is there congruity between the stated philosophical perspective and the research methodology?	+
Is there congruity between the research methodology and the research question or objectives?	+
Is there congruity between the research methodology and the methods used to collect data?	+
Is there congruity between the research methodology and the representation and analysis of data?	+
Is there congruity between the research methodology and the interpretation of results?	+
Is there a statement locating the researcher culturally or theoretically?	−
Is the influence of the researcher on the research, and vice versa, addressed?	−
Are participants, and their voices, adequately represented?	+
Is the research ethical according to current criteria, or for recent studies, and is there evidence of ethical approval by an appropriate body?	+
Do the conclusions drawn in the research report flow from the analysis, or interpretation, of the data?	+
Outcome	8/10

*Note*: The plus symbol ‘+’ indicates a ‘yes’ to the question. The minus symbol ‘−’ indicates a ‘no’ to the question.

Given that the behaviours of nurse leaders were explored in this scoping review, leadership styles were also categorized. Whereas three studies did not report on a specific leadership style (Clark et al., 2011; Lewis & Malecha, 2011; Smith et al., 2018), authentic leadership was examined in two of the included studies (Alkaabi & Wong, 2019; Laschinger & Read, 2016). Furthermore, resonant leadership was examined in one study (Laschinger et al., 2014), as was servant leadership (Neubert et al., 2021). Autocratic, democratic, laissez‐faire, transactional and transformational leadership styles were also investigated in one study (Kaiser, 2017), with transformational leadership being the main focus of investigation in the study. Four key themes were identified: (1) creating a shared vision, (2) educating self and others, (3) fostering accountability and (4) supporting others. Table 4 highlights the key themes reported in this paper.

**TABLE 4:** Key themes

Themes	Specific aspects	Sources
Creating a shared vision	Develop a shared vision of a culture of civility	Clark et al. (2011); Kaiser (2017); Laschinger and Read (2016)
	Establish codes of conducts and policies together with staff to outline clear expectations of acceptable behaviours	Clark et al. (2011)
	Establish healthy practice environments with a focus on civility	Clark et al. (2011)
Educating self and others	Improve own skill and ability as a leader to understand how to appropriately address workplace incivility	Lewis and Malecha (2011); Smith et al. (2018)
	Promote the professional development and abilities of nurses	Kaiser (2017); Laschinger and Read (2016)
	Educate nurses about various conflict styles, conflict resolution and respectful conversations	Clark et al. (2011)
Fostering accountability	Hold oneself and others responsible for acceptable behaviours	Clark et al. (2011)
	Enforce policies and procedures regarding civility in daily workflow	Kaiser (2017)
	Role‐model and reinforce positive behaviours	Clark et al. (2011); Neubert et al. (2021)
Providing support	Focus on building relationships with nurses	Kaiser (2017); Laschinger et al. (2014); Alkaabi and Wong (2019)
	Recognize and reward good work	Kaiser (2017); Laschinger et al. (2014)
	Ask for nurses' input and make decisions accordingly	Kaiser (2017)
	Promote teamwork between nurse and supervisor	Kaiser (2017)

### 3.2 Creating a shared vision

A common theme arising across three of the included studies related to creating a shared vision of a culture of civility. Kaiser (2017) found that including all members of staff, such as nurse managers and staff nurses, in creating a vision was an important aspect of fostering civility. In a qualitative study (Clark et al., 2011), nurse managers and nurse executives reported conducting joint meetings and establishing codes of conducts and policies as a strategy to cultivate civility in the practice setting. Nurse managers focused on fostering healthy practice environments with an emphasis on civility. Likewise, Laschinger and Read (2016) identified a positive relationship between authentic leadership and person–job fit, as well as authentic leadership and civility norms amongst new graduate nurses. Person–job fit describes the six areas of worklife that employees should establish to achieve a stable working relationship with their organization (Leiter & Maslach, 1999). Although these six areas include workload, control, reward, community, fairness and values, it is likely nurse leaders develop a shared vision of civility primarily by addressing community and values. Community relates to the complex social context within an organization, such as social support from co‐workers and supervisors, whereas values describe the ideals, motivations and priorities influencing the relationship between staff and the organization that require alignment for full employee engagement (Leiter & Maslach, 1999).

### 3.3 Educating self and others

In addition to a shared vision, educating self and others was a key theme arising from two of the reviewed studies. Lewis and Malecha (2011) found a negative association between workplace incivility scores and staff nurses' perceptions of the ability of the nurse manager to handle workplace incivility in the general environment. Essentially, staff nurses appreciated nurse leaders who demonstrate a strong ability to manage workplace incivility (Lewis & Malecha, 2011). These findings are consistent with those of Smith et al. (2018) who identified lower levels of reported workplace incivility by staff nurses when there was a positive assessment of the ability, leadership skill and support of their nurse manager. The nurse manager's competence as a leader positively influenced the nurse work environment, which, in turn, was associated with lower levels of workplace incivility (Smith et al., 2018).

In addition to improving their own abilities, two of the reviewed studies (Kaiser, 2017; Laschinger & Read, 2016) highlighted the importance of nurse leaders, showing a genuine interest in staff nurses' development. Helping staff nurses to develop their abilities is likely to involve ensuring reward and an aspect of control over one's work (Laschinger & Read, 2016). Control relates to the degree of autonomy a nurse holds in an organization, whereas reward consists of recognition and incentives that are often associated with professional efficacy and personal accomplishment (Leiter & Maslach, 1999). Conflict management education, which involves teaching nurses how to resolve conflict, problem solve and have respectful conversations, was also identified as an important aspect of practice preparedness to promote and maintain civility (Clark et al., 2011).

### 3.4 Fostering accountability

Three of the included studies addressed how nurse leaders foster accountability. Nurse leaders foster accountability by enforcing policies and procedures in the daily workflow and encouraging staff to do the same (Kaiser, 2017). Role‐modelling and reinforcing positive behaviours are also important components (Clark et al., 2011). One reviewed study identified that nurse leaders who prioritize the needs of staff nurses reduce group‐level workplace incivility by promoting virtuous climate (Neubert et al., 2021). When nurse leaders practised servant leadership, which is strongly related to serving others through role‐modelling and positive interpersonal behaviours, there was a positive association with virtuous climate and a negative association with unit‐level workplace incivility (Neubert et al., 2021).

### 3.5 Providing support

Providing support was a prominent theme across three of the included studies. Support ranged from cultivating relationships with nurses to basing decisions on nurses' input and promoting teamwork. Transformational leadership was strongly associated with all aspects of support (Kaiser, 2017), followed by authentic and resonant leadership (Alkaabi & Wong, 2019; Laschinger et al., 2014). One study identified that close relationships with staff nurses, which is a keynote trait of transformational leadership, enabled nurse leaders to have a positive effect on workplace incivility (Kaiser, 2017). Alkaabi and Wong (2019) identified that workplace incivility by the manager was negatively associated with authentic leadership and, consequently, trust in the manager as perceived by new graduate nurses. The characteristics of authentic leadership, which is largely characterized by the leader possessing a strong moral code, mitigated uncivil behaviours and enhanced civility in the workplace (Alkaabi & Wong, 2019). This supports findings by Laschinger et al. (2014) who identified that resonant leadership, which values the emotional intelligence of the leader, directly influenced job satisfaction amongst staff nurses due to the indirect effects of empowerment, which facilitated lower levels of workplace incivility.

The provision of support was lastly reported by staff nurses as the recognition and reward of good work by nurse leaders in one study of this review (Kaiser, 2017). Reward and recognition were enabled through access to opportunities and resources (Laschinger et al., 2014). In the same study, nurse leaders provided further support by asking for input from staff nurses, basing decisions on this feedback and focusing on creating a strong sense of teamwork (Kaiser, 2017).

## 4 DISCUSSION

There is a general consensus, across the literature, that nurse leaders play a vital role in promoting and maintaining civility amongst nurses in health care organizations. Although this is widely acknowledged, the empirical evidence confirming how nurse leaders accomplish civility is limited. As a result, this scoping review was conducted to identify research regarding how nurse leaders pragmatically achieve civility in the health care setting. The findings suggest that nurse leaders not only manage rudeness when it occurs but also utilize a number of methods that ensure nurses are empowered through a healthy work environment. These predominantly were identified under the key themes of creating a shared vision, educating self and others, fostering accountability and providing support.

Nurse leaders described creating a shared vision and establishing codes of conducts and policies for acceptable behaviours across three of the studies of this review. Broadly speaking, a shared vision directs employees towards shared goals and codes of conducts and policies to establish behavioural norms of respect. These written and verbal contracts are widely regarded to influence organizational culture (Slåtten et al., 2021), with lower levels of workplace incivility associated with a caring culture (Phillips et al., 2018). Employees are more likely to reciprocate respectful behaviours when organizations uphold an environment that demonstrates concern and commitment to them (Memon et al., 2021). A shared vision, in particular, is perceived to be valuable to organizations (Slåtten et al., 2021). However, in order to understand how employees integrate a shared vision successfully, a recent study found that leaders in a hospital setting must be aware of how both personal and environmental‐related factors influence employees (Slåtten et al., 2021). Because employees may be morally disempowered when corporate codes of ethics are written by managers as a directive for employees (Babri et al., 2021), it is unclear how nurse leaders create a shared vision and develop codes of conducts and policies regarding behavioural norms of respect reflective of the wishes and concerns of all nurses.

Enforcing policies and procedures to maintain behavioural norms of respect in the daily workflow, holding oneself and others responsible for acceptable behaviours and role‐modelling positive behaviours were actions found in this review to be reflective of accountability. Accountability is a globally acknowledged concept in nursing that incorporates legal, ethical and professional constructs (Chesterton et al., 2021). Nurses are liable to themselves and others regarding their actions and are also influenced by organizational and societal expectations and boundaries for practice regarding ethical conduct (Chesterton et al., 2021). Although applying these policies and procedures in the daily workflow to ensure nurses are accountable for uncivil behaviours is recommended (Crawford et al., 2019), no research could be sourced that provided empirical evidence on how accountability combats workplace incivility. It is important to acknowledge, however, that civility education, incorporating how to communicate in an assertive manner when conflict occurs, as well as role‐play and cognitive rehearsal that enable nurses to actively practise scenarios of workplace incivility, can be effective (Armstrong, 2018). Practising simulated scenarios of workplace incivility may provide nurses with opportunities to rehearse accountability as it may occur in the clinical setting, but there is scant evidence on how nurse leaders foster accountability to ensure civility in the health care setting.

This review identified that staff nurses' positive perceptions of their nurse leaders' ability to actively manage workplace civility, their leadership capability and the support offered to nursing staff, were associated with lower levels of workplace incivility. Although Laschinger et al. (2009) identified that new graduate nurses' perceptions of their nurse manager's ability were influenced by feelings of empowerment and support, there is little evidence in the literature exploring the manner in which nurse leaders improve their own abilities to manage workplace incivility. Structured leadership development programmes have been described in the literature, but with little focus on workplace incivility. Leadership is considered a ‘common thread in generating change and changing culture in the battle against [workplace] incivility’ (Mikaelian & Stanley, 2016, p. 965). In this review, authentic, servant and transformational leadership styles were associated with lower levels of workplace incivility (Alkaabi & Wong, 2019; Kaiser, 2017; Laschinger & Read, 2016; Neubert et al., 2021). Resonant leadership had an indirect effect on reducing workplace incivility via empowerment (Laschinger et al., 2014). Research supports that employees who work with ethical leaders are less likely to engage in workplace incivility (Taylor & Pattie, 2014). Furthermore, given that environmental factors exemplify the strongest relationship to the antecedents of workplace incivility over individual factors (Han et al., 2022), the absence of strong leadership likely contributes to persistent workplace incivility norms amongst nurses. However, the method in which these leadership styles are learned and enacted in the workplace, in addition to improving their skill in a general sense outside the realm of leadership, to improve the capabilities of the nurse leader has received little attention in the research literature.

The provision of opportunities for staff nurses to develop professionally was described by two of the studies in this review (Kaiser, 2017; Laschinger & Read, 2016). Empowerment has been widely acknowledged as a means to provide opportunities for nurses to advance in their careers. Achieving professional goals encourages a sense of self‐determination and self‐efficacy, which are important aspects of empowerment (Kaiser, 2017). Wing et al. (2015) found that structural empowerment not only negatively correlates with workplace incivility but also contributes to fewer mental health symptoms amongst new graduate nurses. Furthermore, an outcome of a civility intervention identified that nursing staff more readily accessed support and resources due to an increase in awareness regarding worklife issues (Spence Laschinger et al., 2012). However, few studies have identified this area as a means for nurse leaders to promote and maintain civility.

Various conflict styles, conflict resolution and respectful conversations found support in this review as being key components of civility education. The incorporation of education into civility interventions has been found to reduce the prevalence of workplace incivility in health care settings (Leiter et al., 2012). However, Walsh and Magley (2020) argue that, although evaluative studies provide evidence for change, it is unclear as to why these interventions are successful in achieving civility. Studies that are effectiveness oriented aim to uncover why an intervention is successful, which is important when determining how to effectively design and implement tailored civility interventions, as well as understanding the specific components that may impact employees' motivation to learn (Walsh & Magley, 2020). According to Park and Martinez (2022), the effects of risk factors on instigating workplace incivility are greater than the effects of preventative factors, indicating that a dominant focus on minimizing risk factors may aid in promoting civility. Hence, there is limited evidence regarding what specific aspects of tailored civility education are effective.

Finally, in this review of the literature, it was found that nurse leaders provided support to nursing staff by fostering trust, building relationships, and rewarding and recognizing good work. Although these aspects of support are widely recommended to promote civility and combat workplace incivility across the literature, the empirical evidence regarding their effectiveness is somewhat limited. First, trust in the manager has been found to enable nurses to speak more openly about their concerns, share suggestions to enable change and invest in one's work (Wong et al., 2010). In contrast, employees who report poorer trust in their managers are unlikely to recognize workplace incivility as violating norms, as their expectations of their managers were initially poor (Jawahar & Schreurs, 2018). Second, with regard to building relationships, one study in this review (Kaiser, 2017) reported higher levels of workplace incivility when the leader was unconcerned with interpersonal relationships with staff. Structural empowerment has been found to have a partial mediation effect on interpersonal relationships and work engagement (Cziraki & Laschinger, 2015), but the evidence remains limited. Third, reward and recognition are widely regarded as an acceptable method to improve employees' experiences of meaningful work, producing greater reports of well‐being and a positive relationship between work performance and engagement (Salleh et al., 2020). However, Tetrick and Haimann (2014) suggest that, although rewards and recognition are generally received positively by employees, aspects of design and implementation of employee recognition programmes may cause negative consequences, especially when employees feel that they are the centre of attention of evaluation, or simply do not receive recognition. Ultimately, there remains a gap in the literature exploring the effectiveness of the described areas of support that enable nurse leaders to promote and maintain civility in the health care setting.

This scoping review is not without limitations. There were relatively few studies eligible for full‐text review. Seven of the studies were cross‐sectional, restricting any capacity to demonstrate causal relationships between variables. The quality of the remaining qualitative study was relatively weak as there was a potential for selection bias with nurse leaders being recruited at a conference. The conceptual framework of the qualitative study also required empirical testing, being newly adapted from a similar model developed for the academic setting (Clark et al., 2011). In addition, all studies were conducted in North America. Therefore, the strategies and actions of nurse leaders may largely reflect what is acceptable in Western culture, making the findings difficult to generalize to nurse leaders of different cultures.

## 5 CONCLUSION

Workplace incivility requires immediate and targeted action by nurse leaders supported by clear evidence‐based rationales. Creating a shared vision, educating self and others, fostering accountability and providing support were key themes identified in this review reflective of the current strategies and actions employed by nurse leaders to promote and maintain civility in the health care setting. However, our findings indicate a number of gaps in the evidence to support these strategies and actions. Further research is needed to understand how nurse leaders create a shared vision together with nurses, as well as in what manner nurse leaders practise accountability for themselves and for others when unacceptable behaviours occur. Research is also needed to inform how nurse leaders improve their own ability to foster civility in the workplace. Because empowerment is known to impact civility, research into the professional development needs of nurses as it relates to civility requires further attention. Lastly, there is a need to understand what specific aspects of tailored civility education are effective and how nurse leaders employ aspects of support to promote civility. Ultimately, without a strong evidence base to inform practice, workplace incivility will prevail unless nurse leaders equip themselves with the appropriate skills and knowledge to achieve civility for improved outcomes across nurses, patients and organizations.

## 6 IMPLICATIONS FOR NURSING MANAGEMENT

Understanding how to practically achieve civility is essential to performing the role of the nurse leader, regardless of how uncivil or civil a work environment is. A workplace characterized by civility provides a buffer for a range of professional nursing issues, including high turnover rates, burnout, job dissatisfaction and stress. Nurse leaders must be aware that workplace incivility is a serious breach of professional nursing codes of conducts and organizational policies related to acceptable behaviours. However, this awareness needs to be followed by knowledge of the necessary skills and strategies supported by evidence to promote and maintain civility. The findings from this scoping review provide nurse leaders with an overview of how civility is promoted in the health care setting, while also identifying areas of future research required to strengthen the evidence base.

## CONFLICT OF INTEREST

All authors declare no conflict of interest.

## ETHICAL APPROVAL

No ethics review was required for this study.

## Data Availability

The data that support the findings of this study are available from the corresponding author upon reasonable request.
